# Reducing redundancy and enhancing accuracy through a phylogenetically-informed microbial community metabolic modeling approach

**DOI:** 10.1093/bioinformatics/btaf328

**Published:** 2025-07-23

**Authors:** Sepideh Mofidifar, Mojtaba Tefagh

**Affiliations:** Department of Bioinformatics, Institute of Biochemistry and Biophysics, University of Tehran, Tehran, 14176-14335, Iran; Department of Mathematical Sciences, Sharif University of Technology, Tehran, 14588-89694, Iran

## Abstract

**Motivation:**

Metabolic modeling has emerged as a powerful tool for predicting community functions. However, current modeling approaches face significant challenges in balancing the metabolic trade-offs between individual and community-level growth. In this study, we investigated the effect of metabolic relatedness among taxa on growth rate calculations by merging related taxa based on their metabolic similarity, introducing this approach as PhyloCOBRA.

**Results:**

This approach enhanced the accuracy and efficiency of microbial community simulations by combining genome-scale metabolic models (GEMs) of closely related organisms, aligning with the concepts of niche differentiation and nestedness theory. To validate our approach, we implemented PhyloCOBRA within the MICOM and OptCom package (creating PhyloMICOM and PhyloOptCom, respectively), and applied it to metagenomic data from 186 individuals and four-species synthetic community (SynCom). Our results demonstrated significant improvement in the accuracy and reliability of growth rate predictions compared to the standard methods. Sensitivity analysis revealed that PhyloMICOM models were more robust to random noise, while Jaccard index calculations showed a reduction in redundancy, highlighting the enhanced specificity of the generated community models. Furthermore, PhyloMICOM reduced the computational complexity, addressing a key concern in microbial community simulations. This approach marks a significant advancement in community-scale metabolic modeling, offering a more stable, efficient, and ecologically relevant tool for simulating and understanding the intricate dynamics of microbial ecosystems.

**Availability and implementation:**

PhyloCOBRA implementations are available as extensions to the MICOM packages and can be accessed at https://github.com/sepideh-mofidifar/PhyloCOBRA.

## 1 Introduction

In the recent years, the holobiont concept has emerged as a framework for studying the interactions between hosts and their associated microbiota (Zilber-Rosenberg and Rosenberg 2021). Since the stability and composition of the microbiome profoundly influence host-microbiome interactions, it is essential to consider the relationships among microbiota components within holobionts (Simon *et al.* 2019, Zilber-Rosenberg and Rosenberg 2021). Moreover, uncovering the interaction and dependencies within complex microbial communities has long been subject of microbial ecology (Lam *et al.* 2020). Studies have demonstrated that the composition of species in the microbiome exhibits deterministic interactions rather than stochastic neutral forces (Levy and Borenstein 2013, Chen *et al.* 2021). The interplay between metabolic overlap and phylogenetic relatedness has led to competing theories of habitat filtering and niche differentiation. Habitat filtering suggests that phylogenetically similar species dominate microbial communities, while niche differentiation posits that such species are less capable of coexisting due to similar traits and resource overlap (Lam *et al.* 2020). Indeed, community assembly encompasses two seemingly contradictory trends. Species colonizing a site with specific environmental conditions often exhibit similarities in certain phenotypic traits, resulting in trait convergence. However, this ecological similarity can also limit species coexistence within a local community, driving phenotypic trait divergence to minimize competition. Thus, a given set of traits may exhibit both convergence and divergence simultaneously, reflecting the complex dynamics at play (Pillar and Duarte 2010, Machado *et al.* 2021).

Given that major interactions occur at the metabolic level, alternative computational models have been developed to quantify metabolic capabilities of microbial communities (Heinken *et al.* 2013, Bauer and Thiele 2018). Genome scale metabolic modeling has become one of the crucial strategy for predicting the interactions and growth of individuals within a community (Chan *et al.* 2017). While multiple methods have been developed for modeling community metabolism (Zomorrodi and Maranas 2012, Chan *et al.* 2017, Baldini *et al.* 2019, Diener *et al.* 2020), significant limitations remain in elucidating microbial metabolic capabilities and understanding community dynamic (Gottstein *et al.* 2016). One of the key challenges in this modeling is the inherent trade-off between optimizing individual growth rates and maintaining the stability and functionality of the overall community. Previous approaches, such as MICOM and Descriptive OptCom, have attempted to address these trade-offs by incorporating constraints that limit the maximal growth rate of individuals within the community context (Zomorrodi and Maranas 2012, Diener *et al.* 2020). These constraints help to balance the competing demands of individual and community-level growth; however, they do not account for metabolic relatedness within samples. Additionally, genome-scale microbial community modeling approaches often rely on automated or semi-automated reconstruction datasets, such as CarveMe and AGORA, which fail to significantly distinguish the metabolic networks of different organisms. These reconstruction methods frequently depend on various biochemical databases that may contain inconsistencies and incorrect annotations, leading to inaccurate and nonspecific models (Benedict *et al.* 2014). The reliance of CarveMe on an universal model that is manually curated and simulation-ready, limits its applicability to less-studied organisms or those that significantly deviate from the assumptions of the universal model (Machado *et al.* 2018). Furthermore, AGORA’s effectiveness is constrained by the completeness of available genomic data. Incomplete or low-quality genomes can result in gaps in the metabolic models, reducing their predictive power and it is not capable to capture strain-specific variability (Magnúsdóttir *et al.* 2017). Beyond the weaknesses of automated reconstruction methods, intrinsic metabolic similarities among closely related bacteria have also been reported (Ma and Zeng 2004, Russel *et al.* 2017).

Furthermore, transitioning from single-taxon to community models significantly increases complexity, requiring more computational resources to predict community behavior. Dynamic FBA (flux balance analysis) methods, which can better capture the temporal aspects of microbial interactions, are computationally expensive. While steady-state models are more scalable, they may not fully capture the dynamics of microbial communities (Zomorrodi *et al.* 2014, Chan *et al.* 2017, Diener and Gibbons 2023).

In response to these challenges, we introduce PhyloCOBRA, an innovative approach that efficiently integrates metabolic relatedness among taxa while optimizing simulation run times. we merged GEMs (genome scale metabolic models) of phylogenetically related organisms (named as PhyloGEM). Then the simulations were implemented within the MICOM and OptCom frameworks, referred to as PhyloMICOM and PhyloOptCom, respectively. Our results demonstrate that this merging approach yields a more accurate representation of microbial community dynamics compared to conventional methods, offering a significant advancement in the modeling of complex microbial ecosystems.

## 2 Materials and methods

### 2.1 Join models

To generate higher-level GEMs, we first aggregated the abundances of the corresponding lower-level models. The join_models function in MICOM package was then employed to combine phylogenetically related models into a single, unified model. MICOM generated genus and family level datasets using AGORA database through this function. The process of joining models involved integrating the reactions from multiple individual models into a comprehensive framework. A critical component of this integration is the creation of a new biomass reaction that represents the average biomass production capability of all input models. This new reaction was constructed by averaging the coefficients from the biomass reactions of the individual models:


$$v_{bi}^{c}=\frac{1}{N}\sum_{i=1}^{N}v_{\mathrm{bio}}^{i}$$

where $v_{bi}^{c}$ is the flux of the biomass reaction in the merged model resulting from joining N metabolic models with corresponding biomass reactions for *i* = 1, 2, …, *N*. This ensures that biomass production in the merged model accounts for the contribution of all organisms while normalizing by the number of models.

Additionally, the function ensured that only unique reactions from each model were included in the combined model, preventing duplication. By setting this new biomass reaction as the objective, the community model is optimized for this averaged biomass production, reflecting the intra-level relationships of these organisms in growth rate calculations.

Analysis of phylogenetic distance was done using the taxize package in R (Chamberlain and Szöcs 2013). A similarity threshold of 0.6 or higher was used to merge metabolic models, based on the formulation described earlier.

### 2.2 Pearson correlation

The Pearson correlation coefficient is a statistical measure of the strength of linear association between two variables (Sedgwick 2012). In this study, we employed Pearson correlation analysis to evaluate the relationship between the predicted growth rates and the actual replication rates observed across different taxonomic levels. To incorporate phylogenetic relationships, we used two strategies:

1. Pre_PhyloM (Pre_PhyloO): Growth rates were calculated by using MICOM or OptCom approaches after the generation of PhyloGEMs, where phylogenetically related models were merged to reflect intra-level relationships.
2. Post_PhyloM (Post_PhyloO): The analysis involved calculating the weighted mean of growth rates at higher taxonomic levels, derived from MICOM or OptCom simulations.

For both strategies, the actual replication rates were estimated by considering the mean replication rate for each repeated taxonomic level and the weighted mean of the higher taxonomic level within each sample. Additionally, the Cocor package was utilized to perform statistical tests for comparing correlations (Diedenhofen and Musch 2015).

### 2.3 Jaccard distance

Jaccard distance is a statistic used to calculate the dissimilarity and diversity of sample sets. This measurement was performed to assess the dissimilarity both within and across models. The intra-level dissimilarity calculation was performed by measuring the distance between a higher-level category and its related sublevels. For example, in simulations at the order level, the Jaccard distance was calculated based on the dissimilarity between the order models and their corresponding family models. The inter-level dissimilarity calculation involved assessing the distance between each model within a community, providing insight into the diversity of the community as a whole.

### 2.4 Sensitivity analysis

To evaluate the robustness of our method, a sensitivity analysis was conducted by introducing random noise into the abundance data to assess its impact on growth rate predictions. Random noise with a mean of 0 and standard deviations of 0.1, 0.7, and 1.5 was applied to the abundance data. For each level of standard deviation, the noise was introduced five times to each sample, and the mean growth rates across these runs were calculated. Subsequently, the Pearson correlation coefficient was computed between these mean growth rates and the experimental data, enabling us to assess the consistency and stability of our model’s predictions under varying levels of noise.

## 3 Results

### 3.1 Implementation and evaluation of PhyloMICOM performance

**Figure 1.**
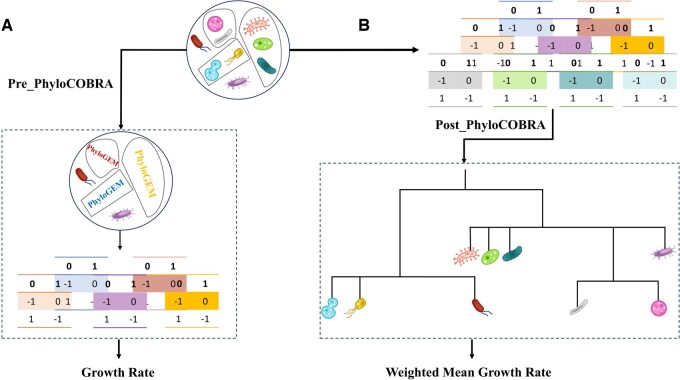
Schematic illustration of the PhyloCOBRA Approach. (A) Pre_PhyloCOBRA: In this approach, PhyloGEM is first performed by merging the genome-scale metabolic models (GEMs) of phylogenetically related organisms. Following this, a microbial community simulation is conducted, and the collective growth rate is calculated. (B) Post_PhyloCOBRA: In this approach, the microbial community simulation is initially performed using individual GEMs. Based on the resulting growth rates, the weighted mean growth rate for phylogenetically related organisms is then calculated, providing insights into their collective behavior.

To validate our framework, we predicted growth rates using three strategies for merging metabolic models: based on taxonomic rank, phylogenetic distance, and metabolic cooperation. For this purpose, we utilized the MICOM package on a balanced cohort of metagenomes from 186 individuals, including healthy participants as well as those with type 1 and type 2 diabetes, along with a sample of a four-species synthetic community. All simulations were conducted on a standard workstation equipped with 10 GB RAM, 150 GB of storage, and 10 CPU threads, demonstrating the method’s computational efficiency. Our study aimed to potentially account complementary and competitive relationship between taxa in the growth rate calculations. Therefore, based on taxonomic rank, in pre-PhyloMICOM we implemented our approach by pooling GEMs of phylogenetically related microorganisms into higher phylogenetic ranks using various datasets provided by MICOM (Fig. 1A). This merging models of phylogenetically related microorganism could impose limitations on the growth rates of sublevel organisms, aligning with niche differentiation theory. Additionally, in the post-PlyloMICOM approach, we evaluated our strategy by calculating the weighted mean of growth rates at higher taxonomic levels derived from MICOM simulations (Fig. 1B). To assess the performance of our simulations, we compared the Pearson correlations of inferred growth rates at each phylogenetic level with the corresponding replication rates provided by MICOM.

**Figure 2.**
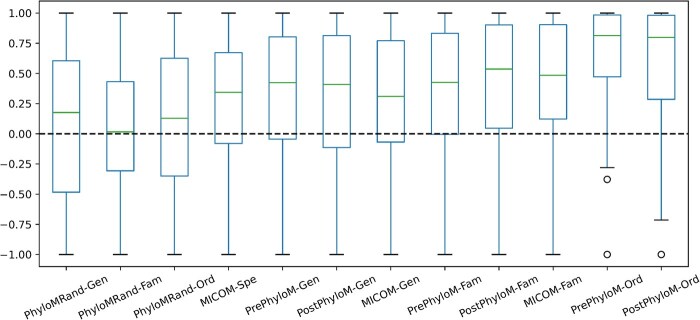
Pearson correlation between replication rates and inferred growth rates for samples based on different taxonomic rank and MICOM approach. The dashed line indicates a correlation coefficient of zero. PhyloMRand-Gen PhyloMICOM random Genus level; PhyloMRand-Fam, PhyloMICOM random family level; PhyloMRand-Ord, PhyloMICOM random order level; MICOM_Spe, MICOM Species level; PrePhyloM_Gen, preprocessed PhyloMICOM Genus level; PostPhyloM_Gen, postprocessed PhyloMICOM Genus level; MICOM_Gen, MICOM genus level; PrePhyloM_Fam, preprocessed PhyloMICOM family level; PostPhyloM_Fam, postprocessed PhyloMICOM family level; MICOM_Fam, MICOM family level; PrePhyloM_Ord, preprocessed PhyloMICOM order level; PostPhyloM_Ord, postprocessed PhyloMICOM order level.

For further validation, we generated random taxonomic groupings based on fixed number of higher phylogenetic ranks within each sample. Assuming that there exist *N* number of family with one related order in a sample, a random taxonomic group for this sample is generated by selecting *N* random families and pooling their related models. We observed that randomly generated taxonomic groups resulted in solutions with lower Pearson correlation (Fig. 2). This finding suggests that the observed correlations are dependent on the inherent structure of the data, indicating that the original phylogenetic relationships are meaningful.

As depicted in the Fig. 2, while Genus family and order levels show moderate positive correlations, PhyloGEM based on order level exhibited significantly better agreement with replication rates (Tables 1–3).

**Table 1.** Williams’ test statistic (1959) for comparing correlations across Genus level^a^.

	PrePhyloM-Fam	PostPhyloM-Fam
PhyloMRand-Fam	0.9738	0.9792
PostPhyloM_Fam	0.942	

a PhyloMRand-Gen, PhyloMICOM random Genus level; PrePhyloM-Gen, preprocessed PhyloMICOM Genus level; PostPhyloM-Gen, postprocessed PhyloMICOM Genus level.

**Table 2.** Williams’ test statistic (1959) for comparing correlations across Family level^a^.

	PrePhyloM-Fam	PostPhyloM-Fam
PhyloMRand-Fam	0.5	0.2978
PostPhyloM_Fam	0.7054	

a PhyloMRand-Fam, PhyloMICOM random family level; PrePhyloM-Fam, preprocessed PhyloMICOM family level; PostPhyloM-Fam, postprocessed PhyloMICOM family level.

**Table 3.** Williams’ test statistic (1959) for comparing correlations across Order level^a^.

	PrePhyloM-Ord	PostPhyloM-Ord
PhyloMRand-Ord	0.0062	0.0356
PostPhyloM-Ord	0.2287	

a PhyloMRand-Ord, PhyloMICOM random order level; PrePhyloM-Ord, preprocess PhyloMICOM order level; PostPhyloM-Ord, postprocess PhyloMICOM order level.

**Figure 3.**
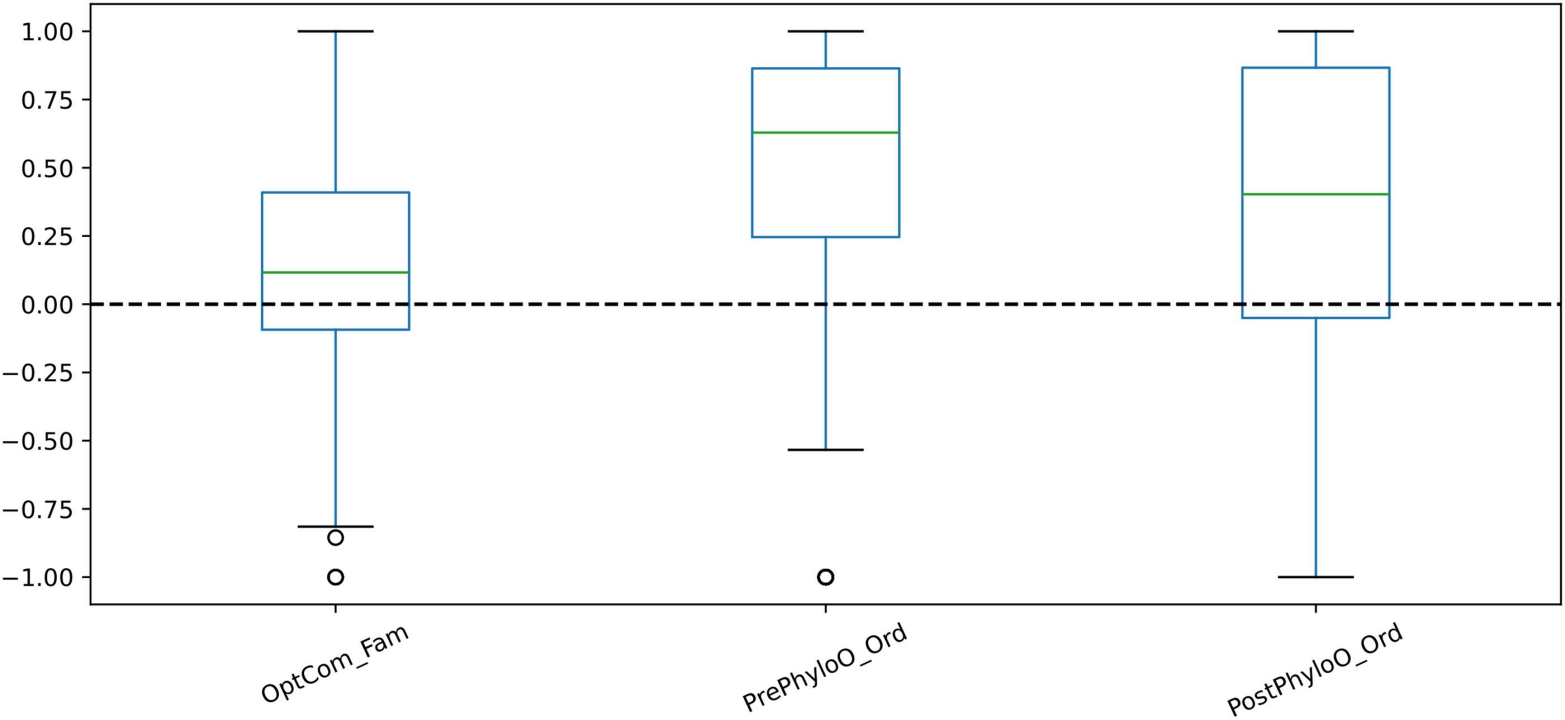
Pearson correlation between replication rates and inferred growth rates for samples based on different taxonomic rank and OptCom approach. The dashed line indicates a correlation coefficient of zero. OptCom_Fam, OptCom family level; PrePhyloO_Ord, preprocessed PhyloOptCom order level; PostPhyloO_Ord, postprocessed PhyloOptCom order level.

Additionally, to assess the compatibility of our method with other approaches, we applied the PhyloGEM approach based on the OptCom method. As depicted in Fig. 3, in line with the observed results from MICOM, PhyloOptCom at the order level demonstrated a better correlation than basic method.

**Figure 4.**
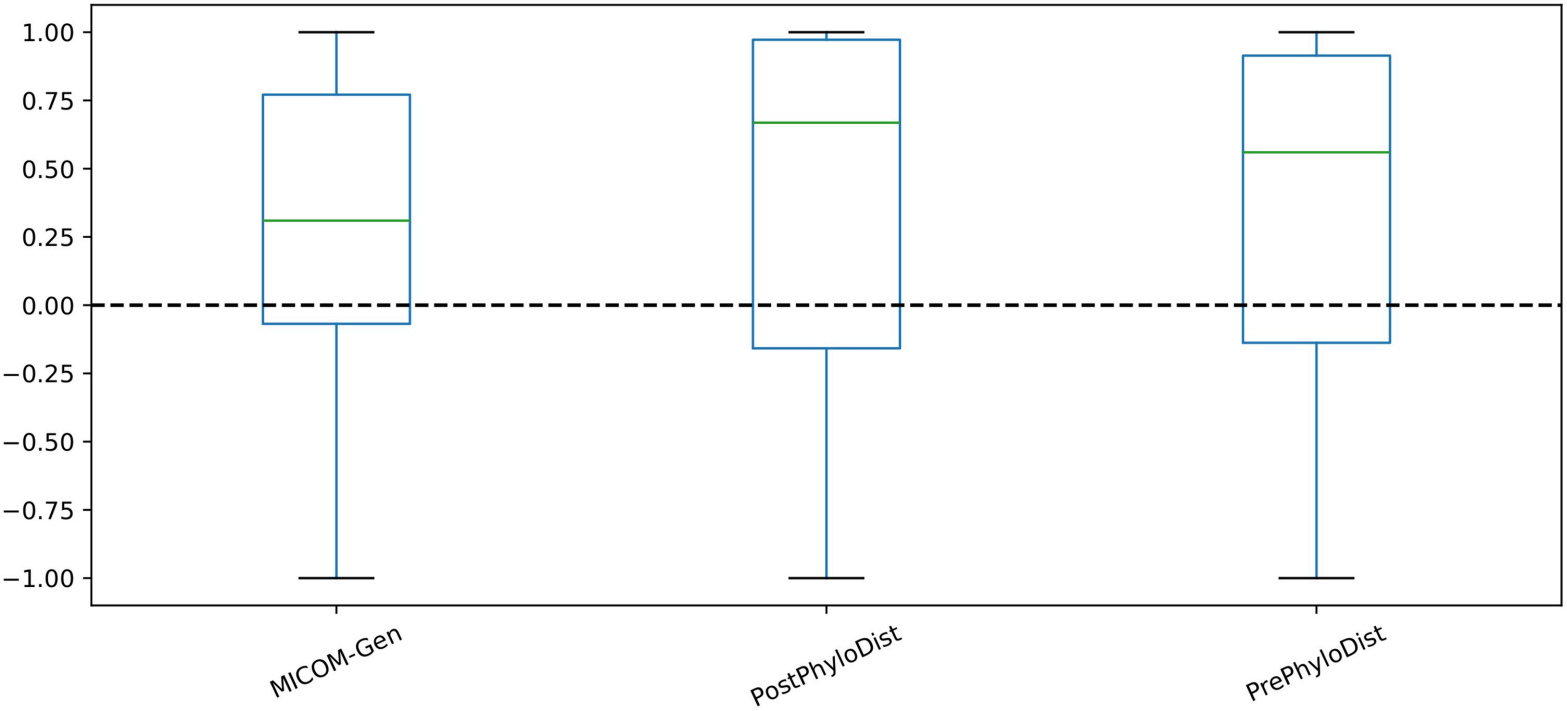
Pearson correlation between replication rates and inferred growth rates for samples based on different phylogenetic distance. The dashed line indicates a correlation coefficient of zero. MICOM_Gen, MICOM genus level; PostPhyloDist, postprocessed PhyloMICOM based on phylogenietic distance; PretPhyloDist, preprocessed PhyloMICOM based on phylogenietic distance.

To explore our strategy based on phylogenetic distances, metabolic models of taxa with a similarity score greater than 0.6 were merged. Although merging metabolic models based on phylogenetic distance showed improved correlation with experimentally measured growth rates compared to the default MICOM package, using taxonomic rank resulted in even better performance (Fig. 4).

To examine the effect of metabolic cooperation on growth rate prediction, we performed simulations based on the four-species synthetic community reported by Hansen *et al.* (2024). This four-membered SynCom (*Pedobacter* sp. D749, *Rhodococcus globerulus* D757, *Stenotrophomonas indicatrix* D763, and *Chryseobacterium* sp. D764) is not phylogenetically related, but the cyclic lipopeptide is inactivated and degraded by the combined action of *R. globerulus* and *S. indicatrix*. Based on this evidence, we performed our simulations by considering all species individually or merging *R. globerulus* and *S. indicatrix* to account for their metabolic cooperations and completeness. Simulations were conducted using both the MICOM and OptCom methods (Table 4). While the correlation for the default approach was negative, merging the models increased the correlation to 0.459.

**Table 4.** Comparison of growth rate predictions between MICOM and PhyloMICOM^a^.

	Relative abundance in percentage			Growth rates				
	Day1	Day2	Day3	Experimental	MICOM	PhyloMICOM	OptCom	PhyloOptcom
Pedobacter sp. D749	9.95	4.77	0.04	−4.955	82.67	0	110.99	0
*Rhodococcus globerulus*	11.12	9.5	22.53	5.705	0	10.24	111.64	10.25
*Stenotrophomonas indicatrix*	71.19	76.02	68.96	−1.115	0	10.24	83.70	10.25
Chryseobacterium sp. D749	7.74	9.71	8.47	0.365	0	0	64.121	0
Correlation					−0.75	0.459	0.05578	0.459

a Relative abundance data were sourced from Hansen *et al.* (2024). For PhyloMICOM and PhyloOptCom metabolic merging was performed between strains with reported metabolic interactions. Growth rates of merged models are highlighted.

### 3.2 Measuring similarity of GEMs based on MICOM and PhyloMICOM

**Figure 5.**
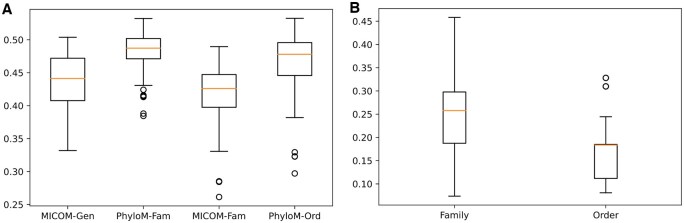
Boxplot of Jaccard distances for metabolic communities using MICOM and PhyloMICOM methods. (A) Intra-level Jaccard distance. (B) Inter-level Jaccard distance. MICOM-Gen, MICOM genus level; PhyloM-Fam, PhyloMICOM family level; MICOM-Fam, MICOM family level; PhyloM-Order, PhyloMICOM order level.

The Jaccard index is one of the most widely used methods to assess the compositional similarity or dissimilarity of species overlap (Chao *et al.* 2005). To compare the redundancy of GEMs in communities generated by MICOM and PhyloMICOM, we calculated both inter-level and intra-level Jaccard distances. For inter-level Jaccard distance, we assessed the dissimilarity within each community generated by MICOM and PhyloMICOM. We observed a higher mean Jaccard distance in PhyloMICOM community models (both family and order levels) compared to MICOM (Fig. 5A). These results indicate the exclusion of highly similar GEM models from samples, thereby reducing redundancy.

For intra-level Jaccard distance, we measured the dissimilarity between GEMs at a particular level and their corresponding higher-level GEMs. As illustrated in Fig. 5B, the Jaccard distance between the GEMs of each order and their related families in the PhyloMICOM order level indicates a lower degree of similarity than in the PhyloMICOM family level. This suggests a greater degree of differentiation and less overlap between the PhyloGEMs at the order level, reflecting increased diversity and reduced redundancy.

### 3.3 Simulation run time

**Figure 6.**
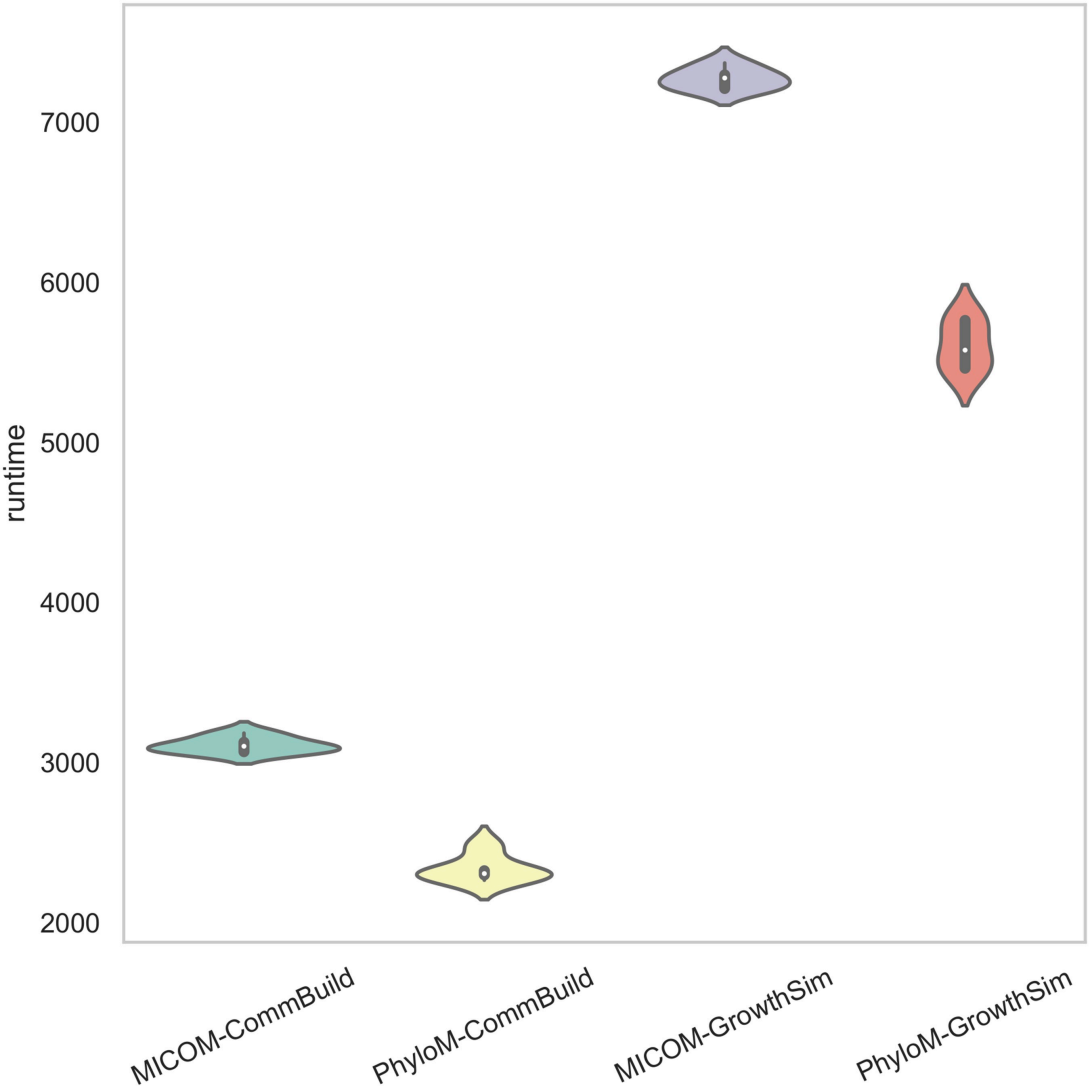
Comparison of runtime performance for microbial community generation and growth rate calculation between MICOM and PhyloMICOM methods. MICOM-CommBuild, MICOM community building; PhyloM-CommBuild, PhyloMICOM community building; MICOM-GrowthSim, MICOM Growth rate simulation; PhyloM-GrowthSim, PhyloMICOM Growth rate simulation.

To compare the runtime performance of each method for building models, we used the pytest benchmark fixture (https://github.com/pytest-dev/pytest). The simulation times, which included both the construction of community models and the calculation of growth rates, are presented in Fig. 6. As shown in this figure, the PhyloMICOM method exhibited significantly shorter runtimes compared to the default MICOM model generation. In MICOM, building community models involves several preprocessing steps for each individual model. These steps include adding suffixes (e.g. the name of the organism) to metabolites and reactions, adding exchange reactions, and incorporating the objective of the model into the community framework. This preprocessing ensures that each model is uniquely identifiable and functionally integrated into the community simulation. As the PhyloMICOM approach reduces the number of individual models by joining closely related organisms into a single model, the total number of models requiring preprocessing effectively decreases. Consequently, the simulation runtime is profoundly reduced because fewer models need to undergo these computationally intensive preprocessing steps.

For a more precise comparison of runtime between different methods, three samples were selected based on varying numbers of taxa. As shown in Table 5, PhyloMICOM achieved approximately half the simulation runtime compared to the default MICOM and OptCom approaches. This reduction is particularly significant for OptCom, as its growth rate calculation is inherently more time-consuming. As the community models are compressed in PhyloMICOM, loading the models and solving the optimization problems become significantly more efficient. Fewer models mean fewer data to load and manage, which reduced the overhead in memory and computational resources. This efficiency led to shorter overall simulation times, making the PhyloGEM method more time-effective for large-scale community modeling and time-consuming optimization methods.

**Table 5.** Runtime comparison of different community simulation methods across samples^a^.

Ssample_id	Total_ taxa	MICOM_ community	Phylo_ community	Growth_ MICOM	Growth_ PhyloMICOM	Growth_ OptCom	Growth_ PhyloOptCom
ERR321172	12	25.04	17.15	5.81	3.68	233.32	89.86
ERR260139	19	58.45	38.96	11.11	7.63	1151.52	488.14
ERR414351	41	112.55	69.465	15.33	11.98	4613.11	1958.74

a Columns represent the total number of taxa per sample and the runtime (in seconds) for each method. Total_taxa, total number of taxa per sample; MICOM_community, created community based on MICOM approach; Phylo_Community, created community based on PhyloGEM approach; Growth_MICOM, growth rate calculation based on MICOM; Growth_Phylo_MICOM, growth rate calculation based on PhyloMICOM, Growth_OptCom, growth rate calculation based on OptCom; Growth_Phylo_OptCom, growth rate calculation based on PhyloOptCom.

### 3.4 Sensitivity analysis

**Figure 7.**
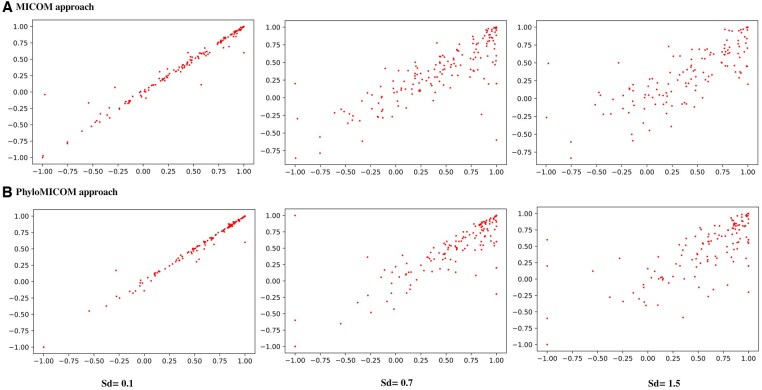
Sensitivity analysis using Gaussian distribution with mean value zero and standard deviation 0.1, 0.7, and 1.5. (A) MICOM approach, (B) PhyloMICOM approach.

We performed sensitivity analysis by introducing random noise with a Gaussian distribution (mean = 0, standard deviations = 0.1, 0.7, 1.5) to generate varying abundances of taxa. As depicted in Fig. 7, the correlation with a noise level of 0.1 remained stable, demonstrating significant robustness in both the MICOM and PhyloMICOM methods. This stability indicates that both methods can reliably handle minor variations in abundance without substantially affecting their growth rate predictions.

However, when the random noise level was increased to 0.7, both models exhibited increased sensitivity, with this effect being more noticeable in the MICOM method than in PhyloMICOM. The higher noise levels caused greater fluctuations in the predicted growth rates, underscoring MICOM’s vulnerability to variability in the input data. Although both methods showed significant sensitivity to a random noise level of 1.5, the impact was more severe in MICOM.

### 3.5 Taxon knockout analysis

**Figure 8.**
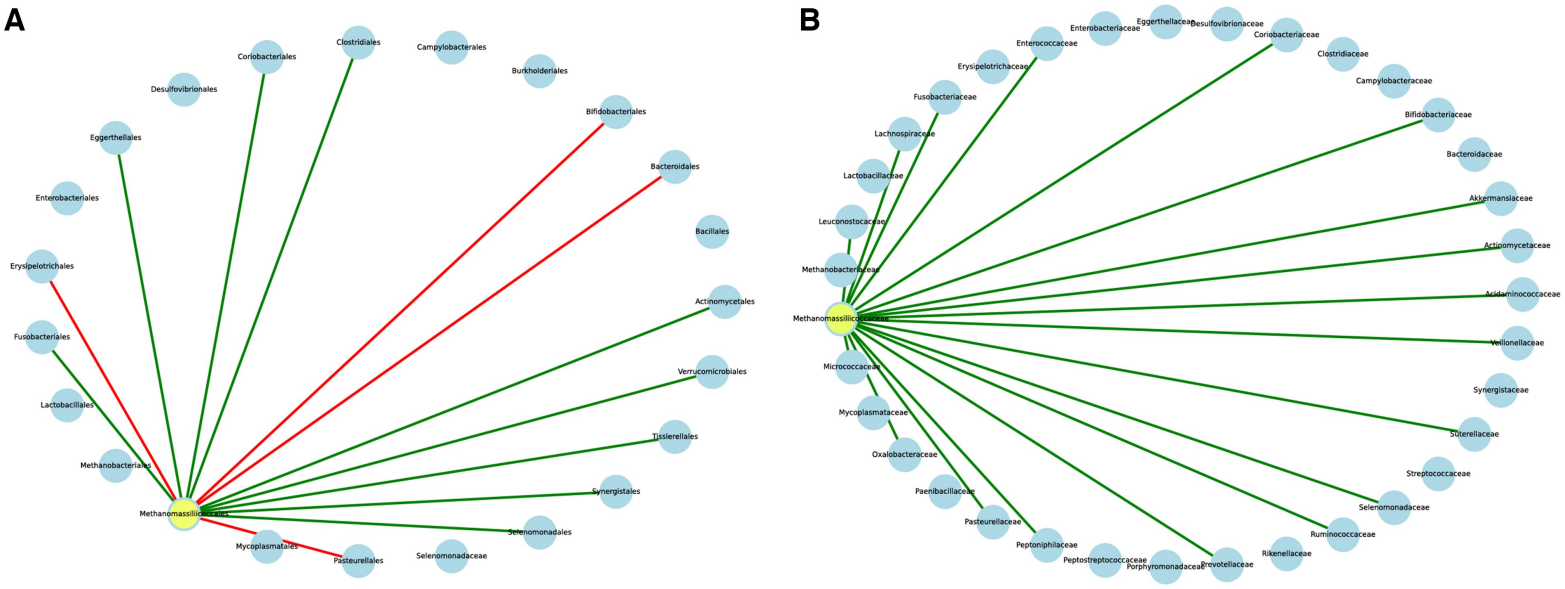
Growth rate interactions between Methanomassiliicoccales and other taxon within samples estimated by taxon knockouts. (A) MICOM approach, (B) Post-PhyloMICOM approach. The color of the edges indicates change of growth rate and type of interaction. Red edges denote competition where removal of one genus increases the growth rate of the other, and green edges denote cooperation where the removal of one genus lowers the growth rate of the other.

We performed a taxon knockout analysis using the MICOM approach. Briefly, for each genus in each sample, a knockout simulation was conducted, and the resulting changes in growth rates for all remaining genera in the sample were tracked. Specifically, in the Methanomassiliicoccales order, the knockout simulation revealed differing interactions between taxa when comparing the MICOM and PhyloMICOM approaches (Fig. 8). While the MICOM model may oversimplify interactions within the Methanomassiliicoccales order by primarily highlighting positive growth impacts, PhyloMICOM captured both positive and negative impacts. Previous studies have shown that the Clostridiales and Enterobacterales orders are involved in trimethylamine (TMA) production (Romano *et al.* 2015, Liu and Dai 2020), while the Methanomassiliicoccales order is involved in TMA consumption (Borrel *et al.* 2017, De la Cuesta-Zuluaga *et al.* 2021, Zhou *et al.* 2021), potentially explaining the positive correlation between these orders. Both MICOM and PhyloMICOM predicted a positive relationship between the Methanomassiliicoccales and Clostridiales orders (specifically Ruminococcaceae in MICOM). Furthermore, studies have reported a negative correlation between Bifidobacteriales and TMAO levels, suggesting that Bifidobacteriales could potentially reduce plasma TMAO levels (Manor *et al.* 2018, Wang *et al.* 2022). This would indicate a negative correlation between Methanomassiliicoccales and Bifidobacteriales. While MICOM was unable to represent this negative relationship, PhyloMICOM successfully captured it, providing a more accurate depiction of microbial interactions.

### 3.6 Interaction analysis

**Figure 9.**
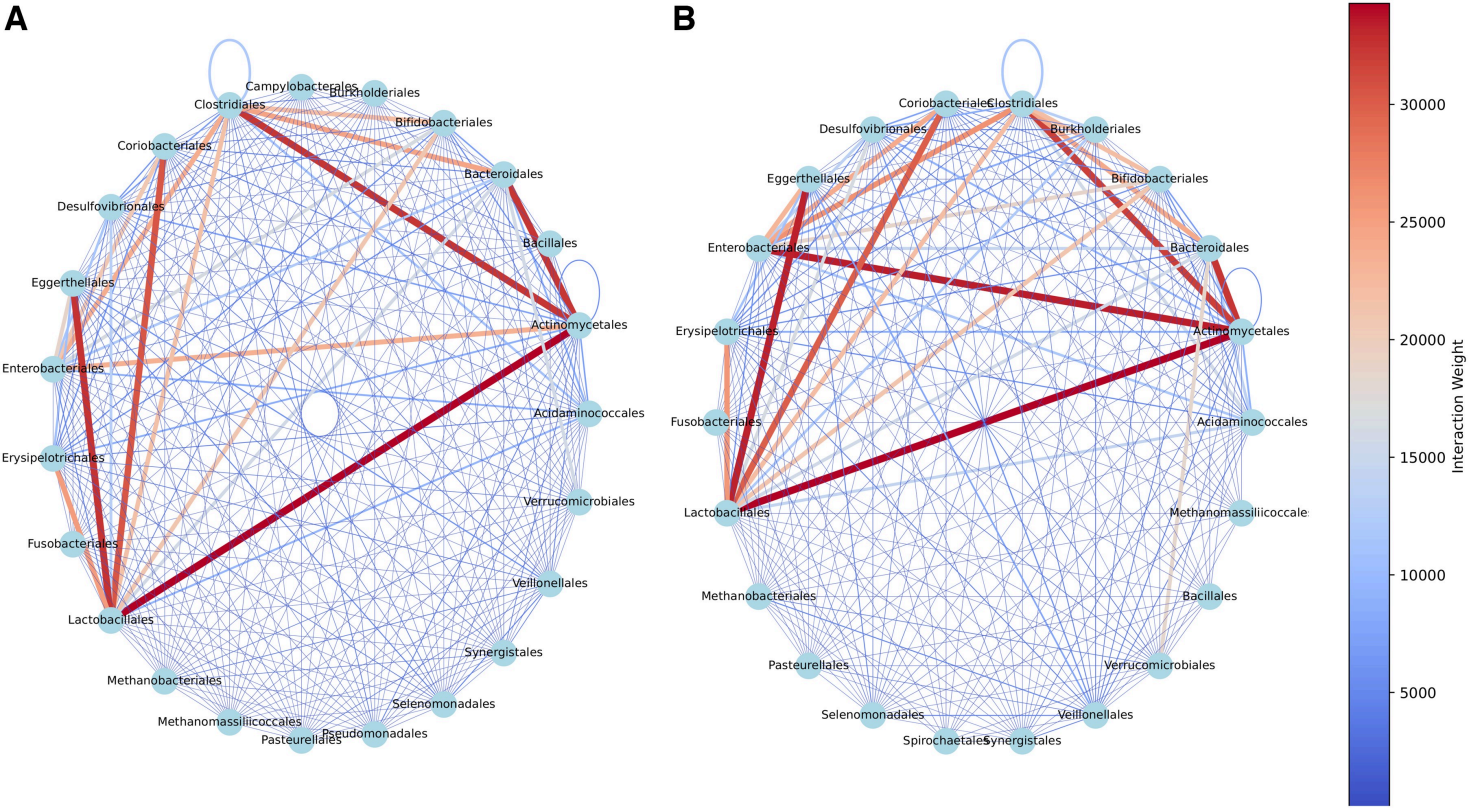
Microbial interaction network. (A) Healthy samples, (B) T2D samples.

One of the major concerns in the microbial community is exploring the interaction of individuals within samples (Datry *et al.* 2017, Mariani *et al.* 2024). To this aim, we imployed the previously mentioned method of detecting de novo interactions (Mariani *et al.* 2024). Briefly we detected the interactions of individuals based on presents of block metabolites in other individuals within each sample. The final graphs were obtained by merging all results based on healthy and T2D status and weighting the occurrence of them in samples. As it is depicted in Fig. 9, the difference of healthy and T2D sample interaction is present of stronger interaction between Actinomycetales and Enterobacteriales in T2D samples. Overabundance of Enterobacteriales is often reported in association with gut dysbiosis, inflammation, and endotoxin production, which are characteristic features of metabolic disorders like T2D (Chindelevitch *et al.* 2012, Daoud 2017, Hsieh *et al.* 2024). This family, particularly Gram-negative bacteria, contributes to lipopolysaccharide (LPS) production, a key driver of metabolic endotoxemia, which has been implicated in insulin resistance and systemic inflammation (Chan *et al.* 2022). The presence of Actinomycetales in this interaction suggests a potential metabolic exchange, as some of the detected metabolites linking these two orders are related to fatty acid biosynthesis, which plays a crucial role in LPS production. This interaction may facilitate the proliferation and persistence of Enterobacteriales in the dysbiotic gut environment of T2D, further exacerbating inflammation and metabolic dysfunction. The weakness of this interaction in healthy individuals suggests a balanced gut microbiome may prevent the opportunistic expansion of pro-inflammatory bacteria, thereby maintaining microbial homeostasis and gut health.

## 4 Discussion

The widespread use of DNA sequencing in microbial community studies, has significantly deepened our understanding of these complex ecosystems (Gilbert *et al.* 2018). Metabolic modeling has become as a critical tool for making quantitative predictions, such as the growth rates of individuals within these communities (Diener and Gibbons 2023). However, accurately predicting growth rates in microbial communities presents substantial challenges, primarily due to the inherent trade-offs between individual and community growth rates. These trade-offs arise because the metabolic activities that benefit individual microorganisms may not always align with those that optimize the overall community growth and stability (Diener *et al.* 2020). Addressing these conflicting demands requires advanced modeling approaches that can account for the intricate interactions and dependencies within the community. In this study, we introduced PhyloCOBRA, a novel approach that balances cooperative and competitive interactions within metabolic model communities. This dependency of organisms can be assessed through various methods. In this study, we introduced PhyloCOBRA, a novel approach to incorporate phylogenetic relatedness within metabolic model communities. Unlike previous models that may have oversimplified these dynamics (Zomorrodi and Maranas 2012, Diener *et al.* 2020), PhyloCOBRA integrates phylogenetic relationships among taxa to improve the accuracy of community simulations. This framework is also extendable to incorporate phylogenetic distance and metabolite cooperation.

The PhyloCOBRA approach is consistent with niche differentiation theory, as it calculates the mean growth rate of closely related species, thereby constraining their growth rates based on phylogenetic relatedness (Lam *et al.* 2020). Additionally, this methodology aligns with the concepts of nestedness and hierarchy theory. In these theory, smaller communities (formed by joining GEMs of closely related taxon in our study) exist within a larger community, with each level contributing to the overall dynamics and stability of the ecosystem (Datry *et al.* 2017, Congreve *et al.* 2018). The concept of a nested pattern is not only present in the gut microbiome (Cobo-López *et al.* 2022) but also in other complex systems, from ecological to economic communities (Mariani *et al.* 2024).

Moreover, as discussed earlier, phylogenetically related organisms exhibit significant similarities due to intrinsic factors and limitations in model reconstruction methods. Also, a recent study highlighted that even when automated reconstruction approaches use the same genomes, the resulting GEMs can differ in the number of genes, reactions, and metabolic functionalities, indicating that they may not accurately represent specific strains (Hsieh *et al.* 2024). This inconsistency has led to the development of algorithms like MetaMerge, which reconcile pairs of existing metabolic models of an organism into a single, unified model (Chindelevitch *et al.* 2012). Such inconsistencies can also introduce bias when predicting metabolite interactions using community GEMs (Hsieh *et al.* 2024). To address this issue in microbe community modeling, we used PhyloGEM, which merges the metabolic models of closely related organisms to reduce redundancy while calculating the mean coefficients for biomass reactions. This strategy aligns with the concept of multicollinearity in statistics. When a strong correlation exists between two or more independent variables in a regression model, the phenomenon of multicollinearity appears (Daoud 2017). This issue can lead to unstable and unreliable parameter estimation, complicating the interpretation of individual effects. One common way to address this problem is to drop the redundant variable (Lin 2008, Chan *et al.* 2022).

The issue of multicollinearity is also present in RNA-seq data analysis, where highly correlated genes can lead to critical biological problems, such as the selection of non-causal groups for a phenotype. Clustering in RNA-seq data groups genes based on expression patterns, effectively managing redundancy and highlighting significant relationships (McDermaid *et al.* 2019, Tian *et al.* 2019). Similarly, in the principles of Principal Component Analysis (PCA), correlated variables are combined into principal components, reducing dimensionality while preserving variance (Hasan and Abdulazeez 2021). Similarly, in community-scale metabolic models, multicollinearity arises from the high similarity among individual GEMs. To address this issue, we developed PhyloCOBRA, which merges GEMs of closely related organisms, thereby reducing redundancy within the community model. This merging process is analogous to the clustering and dimensionality reduction techniques used in RNA-seq analysis and PCA, as it consolidates similar metabolic functions and minimizes overlapping variables. As clustering methods in transcriptomics can rely on various strategies, the merging of metabolic models can also be approached through different criteria, with taxonomic rank being one of the primary focal points in our framework.

This study presents PhyloCOBRA, a novel approach to microbial community modeling that integrates phylogenetic relationships to improve simulation accuracy and efficiency. PhyloGEM effectively reduces the impact of highly correlated variables, thereby enhancing the robustness and reliability of community model predictions. Although PhyloCOBRA offers a more stable and meaningful representation of microbial community dynamics, it may obscure the unique contributions of individual species within the community and may affect accuracy in communities where unique metabolic functions are strain dependent. Furthermore, while phylogenetic relationships provide valuable evolutionary context, they do not always translate directly into functional metabolic similarity. For example, closely related species may inhabit vastly different environments and evolve distinct metabolic pathways, while distantly related species in similar ecological niches may acquire analogous metabolic traits (Yang 2021, Pontes *et al.* 2024). As such, merging metabolic models solely based on taxonomic rank may not always capture sample specific metabolic diversity. In this work, our simulation approach was focused on taxonomic rank, which provides a general framework but may overlook fine-scale metabolic differences. Therefore, we propose for further research to assess the robustness and applicability of this method across diverse microbial systems when using phylogenetic distance, particularly by exploring different hyper parameters and strategies for its calculation. Future work could also investigate the extensibility of this approach to other microbial kingdoms, such as fungi and archaea.

## Data Availability

All the information and implementation of algorithms can be found in the GitHub repository: https://github.com/sepideh-mofidifar/PhyloMICOM. This repository contains the implementation of PhyloMICOM and scripts for analyzing MICOM and PhyloMICOM communities.
